# Knitting Elastic Conductive Fibers of MXene/Natural Rubber for Multifunctional Wearable Sensors

**DOI:** 10.3390/polym16131824

**Published:** 2024-06-27

**Authors:** Zirong Luo, Na Kong, Ken Aldren S. Usman, Jinlong Tao, Peter A. Lynch, Joselito M. Razal, Jizhen Zhang

**Affiliations:** 1 Chinese Agricultural Ministry Key Laboratory of Tropical Crop Product Processing, Agricultural Product Processing Research Institute, Chinese Academy of Tropical Agricultural Sciences, Zhanjiang 524001, China; luozirong3@163.com (Z.L.); na.kong@deakin.edu.au (N.K.); 2 School of Life and Environmental Science, Centre for Sustainable Bioproducts, Deakin University, Geelong, VIC 3216, Australia; 3 Institute for Frontier Materials, Deakin University, Geelong, VIC 3216, Australia; k.usman@deakin.edu.au (K.A.S.U.); peter.lynch@deakin.edu.au (P.A.L.); joselito.razal@deakin.edu.au (J.M.R.)

**Keywords:** MXene, natural rubber, knitting textile, conductive fiber, elastic fiber, wearable sensors

## Abstract

Wearable electronic sensors have recently attracted tremendous attention in applications such as personal health monitoring, human movement detection, and sensory skins as they offer a promising alternative to counterparts made from traditional metallic conductors and bulky metallic conductors. However, the real-world use of most wearable sensors is often hindered by their limited stretchability and sensitivity, and ultimately, their difficulty to integrate into textiles. To overcome these limitations, wearable sensors can incorporate flexible conductive fibers as electrically active components. In this study, we adopt a scalable wet-spinning approach to directly produce flexible and conductive fibers from aqueous mixtures of Ti_3_C_2_T_x_ MXene and natural rubber (NR). The electrical conductivity and stretchability of these fibers were tuned by varying their MXene loading, enabling knittability into textiles for wearable sensors. As individual filaments, these MXene/NR fibers exhibit suitable conductivity dependence on strain variations, making them ideal for motivating sensors. Meanwhile, textiles from knitted MXene/NR fibers demonstrate great stability as capacitive touch sensors. Collectively, we believe that these elastic and conductive MXene/NR-based fibers and textiles are promising candidates for wearable sensors and smart textiles.

## 1. Introduction

Fiber-shaped stretchable sensors possess a unique combination of electrical conductivity and resilience to mechanical deformations, offering great potential in applications such as healthcare, movement detection, and human–machine interaction [1,2]. Their main component—stretchable conductive fibers—however, needs to have the ability to cover movable and arbitrarily shaped objects, and must be ubiquitously integrated into fabrics or even directly attachable to the human skin for wearable device applications [3,4]. Numerous efforts have been made to develop stretchable fibers with excellent conductivity using a variety of materials encompassing carbonaceous nanomaterials (graphene and graphene derivatives, carbon nanotubes, and carbon black) [5,6], metal-based nanomaterials (silver nanoparticle and nanowires) [7,8], conducting polymers [9] and semi-conductor materials such as silicon [10]. The current mainstream strategy to fabric stretchable conductive fibers is categorized in two ways: (i) manufacturing elastic architectures using conducting nanomaterials (such as coiled carbon nanotube yarns [11]) and/or knitting fabric that can be easily stretched in different directions [12,13] and (ii) using elastomeric polymer or hydrogel as a stretchable host to integrate conductive guests [14]. Both engineering novel elastics architectures with non-stretchable fibers and introducing conductive fillers to elastomer have considerable advantages to satisfy the required mechanical properties, while the latter approach shows great potential in industrial-scale textile manufacturing processes which can be realized through spinning, coating, printing, plating and injecting methods [2,14,15].

MXenes, a class of two-dimensional materials composed of transition metal carbides and nitrides, have emerged as promising candidates for crafting functional fibers. Their notable high electrical conductivity and flexible processing attributes make them ideal for innovative applications [16]. In our previous work, additive-free MXene in dispersion was assembled into fibers through wet-spinning [17]. The electrical conductivity of these fibers reached up ~7750 S cm^−1^, suggesting it is a good candidate for e-textiles [17]. However, pure MXene fibers exhibit a poor strain-to-break of only ~1.7%, and cannot be directly knitted or woven into textiles using industrial-scale machines that typically require high mechanical integrity. In order to achieve stretchable functional MXene-based fibers, appropriate polymeric matrices such as polyurethane (PU) [14,18,19,20], polylactic acid (PLA) [21], polyvinyl alcohol (PVA) [22,23], and polyvinylidene fluoride (PVDF) [24] have been employed as the soft substrate of the composite fibers. For example, in the work of Kang et al. [25], the incorporation of a mere 1.0 wt.% of few-layer MXene into epoxy nanocomposites resulted in a significant enhancement of the material’s thermal properties. The thermal conductivity of the composite fibers was elevated to 0.587 W/m·K, marking an impressive 141.3% increase over the un-reinforced samples. This finding underscores the potential of MXenes to substantially improve the performance of composite materials. Using wet-spinning, Seyedin et al. [14] produced a continuous spool of Ti_3_C_2_T_x_/PU hybrid fibers that exhibited the lowest percolation threshold (≈1 wt.%) among MXene-based composites and high Young’s modulus (≈20.3 GPa) among PU-based composite fibers. These examples of MXene/polymer-based composites demonstrate great potential in flexible electronics ascribed to their reliable conductivity and durable mechanical stretchability. Nevertheless, the spinning dopes of these polymers-based MXene composites use organic solvents such as dimethyl formamide, dimethyl sulfoxide and dichloromethane, which are then required to be removed after the molding process, as the presence of remaining organic molecules between the flakes dramatically increases contact resistance. Due to these constraints, and considering the scalability of the processing method, the use of aqueous blends of polymer and MXene has recently been regarded as a more straightforward alternative to prepare spinning dopes.

Natural rubber (NR) latex, a homogeneous colloidal system composed of *cis*-1,4-polyisoprene units, is an ideal matrix for manufacturing flexible nanocomposites owing to its high elasticity, outstanding fracture resistance, good fatigue tolerance and facile processibility [26,27,28,29]. Various nanomaterials, such as nano-silica, carbon nanotubes, graphene, and nanocellulose, have demonstrated dispersibility with this natural aqueous dispersion, providing properties including, but not limited to, light emission/absorption, electrical conductivity, sound harvesting and electromagnetism. Ascribed from their polar surface functionalities (-Oh, =O and/or -F), MXene nanosheets in particular have shown excellent compatibility with NR particles [28,29,30,31,32]. This natural aqueous polymer/nanomaterial system has proved to be a promising candidate for developing flexible, lightweight and conductive composites at a large scale. However, recent efforts on MXene/NR composites have focused on utilizing films for electromagnetic interference (EMI)-shielding purposes, while other applications with the benefit of having an elastic matrix (e.g., strain-sensing fibers) have remained rarely explored [31]. Additionally, providing good mechanical performance and electrical conductivity to MXene-based elastic fibers still necessitates an overall understanding and detailed analyses of the synergy between conductive fillers and polymer hosts during stretching.

**Figure 1:**
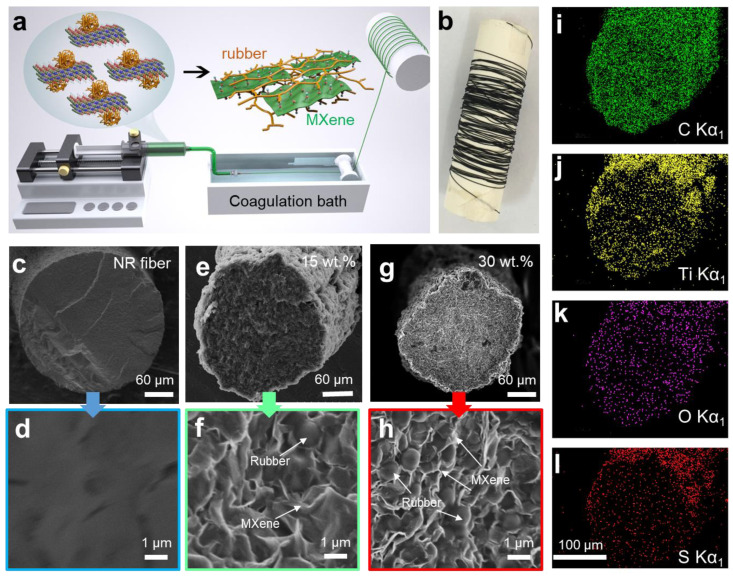
(**a**) Schematic illustration of the custom-built wet-spinning set-up used in this work to produce MXene/NR fiber. (**b**) Digital photograph of a 5 m long MXene/NR fiber which has been continuously collected on a spool. (**c**–**h**) SEM images of the cross-section of pure NR fiber, MXene/NR fiber with 15 wt.% and 30 wt.% content of small MXene (S-MX). (**i**–**l**) EDX elemental maps of carbon, titanium, oxygen, and sulfur of S-MX-15/NR fiber.

Herein, we produced MXene/NR fibers through direct wet-spinning of their aqueous mixtures, as illustrated in Figure 1a. The obtained continuous MXene/NR fiber (>tens of meters in length) demonstrated good stretchability and suitable conductivity ascribed to the synergy between the electrically conductive MXene network and elastic rubber chains. By exploring the influence of MXene sheet sizes, coagulation baths and MXene loading on the composite fiber properties, spinning condition and fiber composition were tailored to the mechanical and electrical requirements for stretchable motivation sensors. Additionally, the resulting elastic MXene/NR fibers are knittable into circular textiles of good flexibility and stable conductive connection suitable for capacitive touching sensors. Overall, these qualities demonstrate the capacity of MXene/NR fibers as materials for smart textiles and textile-based human–machine interfaces.

## 2. Materials and Methods

**Synthesis of Ti_3_C_2_T_x_ MXene.** The synthesis procedure followed our previous publications [33,34]. Ti_3_C_2_T_x_ MXene was synthesized through the selective etching of aluminum layers from the Ti_3_AlC_2_ MAX phase, with a particle size of less than 40 μm, sourced from Jilin 11 Technology Co., Ltd., Changchun, China. The etching process involved preparing a solution by dissolving 1.6 g of lithium fluoride (purity: 99%, supplied by Aladdin Scientific Corp., Riverside, CA, USA) in 20 mL of 9 M hydrochloric acid (HCl), followed by stirring for 5 min. Subsequently, 1 g of Ti_3_AlC_2_ powder was introduced into the etching solution. The reaction was maintained for 24 h at a temperature of 35 °C. Post reaction, the acidic dispersion underwent multiple centrifugation cycles at 3500 rpm for 5 min each, using an Eppendorf 5810R centrifuge (Eppendorf North America, CT, USA), until the pH of the mixture approached neutrality (pH~6), indicating self-delamination. The final step involved centrifuging the dispersion at 1500 rpm for 30 min to segregate the delaminated MXene flakes and any residual MAX phase particles. This method ensured the successful production of few-layer MXene.

To isolate large Ti_3_C_2_T_x_ flakes, referred to as L-MX, the synthesized MXene ink was centrifuged at 4500 rpm for 30 min. The sediment, primarily composed of large flakes, was then redispersed in water. Conversely, small Ti_3_C_2_T_x_ flakes, or S-MX, were generated by subjecting the synthesized Ti_3_C_2_T_x_ to probe sonication using a Fisher Scientific 505 Sonic Dismembrator (Thermo Fisher Scientific Inc., Waltham, MA, USA) at 500 W. This process lasted 20 min with a pulsing regime of 8 s on and 2 s off at 50% power. An ice bath was employed throughout to mitigate any thermal effects. Following sonication, the mixture was centrifuged at 3500 rpm for 10 min, and the supernatant, enriched with small flakes, was collected. This methodical approach enabled the separation of MXene flakes by size, facilitating their use in subsequent applications.

**Prevulcanization of natural rubber latex:** A conventional prevulcanization formulation was used to prepare the prevulcanized NR (PNR) latex [35,36]. Natural rubber latex (60 wt.%) was mixed with the vulcanization ingredients listed in Table S1 at room temperature. The prevulcanization process was carried out at 50 °C for 30 min along with continuous stirring. Then, the prevulcanized NR (PNR) latex was cooled down to room temperature and stored at 4 °C in a freezer before further experiments.

The chloroform number test is a method used to evaluate the degree of prevulcanization of latex by coagulating a sample with an equal volume of chloroform. After mixing, the sample is allowed to sit for 2–3 min, after which the resulting coagulum is inspected and rated based on its texture. In this instance, freshly prepared PNR latex was subjected to the chloroform number test and was determined to be lightly vulcanized as per the described procedure. This assessment is crucial for ensuring the desired properties of the latex in its end-use applications.

**Knitting MX/NR Fibers into Textiles:** MX/NR fibers were knitted on a custom-designed circular knitting machine with 6 hooks and diameter of 10 mm. The fiber was hooked and pulled down to make a knot with itself and this process was repeated as the knitting machine rotated. The knitted fabric passed through the middle hole and was collected under the knitting machine. The knitting machine was connected to a stepper motor to control the rotating rate (1 to 10 cycles per second).

**Characterization:** A HITACHI S4800 scanning electron microscope (Hitachi High-Technologies, Japan) was employed to examine the morphology and cross-sectional structure of MXene/NR fibers. The thickness of MXene flakes was quantified using atomic force microscopy (AFM), specifically a FlexAFM equipped with Nanosurf C3000 software (version 3.10.5), operating in air tapping mode. For AFM analysis, samples were prepared by depositing a diluted MXene dispersion onto silicon wafers. The mechanical properties of the yarns were evaluated using a Tensile Tester TM2101-T5, featuring a 50 N load cell. During testing, yarns were secured with a 1 cm gap between grips, and data on strain and stress were collected at a rate of 1 mm/min. The zeta potential of Ti_3_C_2_T_x_ MXene solutions with varying natural rubber latex (NRL) concentrations was measured using a Malvern Zetasizer Nano ZS90 (Malvern Instruments, Reino Unido, UK), which also facilitated dynamic light scattering (DLS) assessments to determine the size distribution of Ti_3_C_2_T_x_ MXene and NR particles. X-ray diffraction (XRD) patterns for the MAX phase, MXene, and assorted MXene films were acquired with a Rigaku SmartLab 9 kW X-ray powder diffractometer (Rigaku Corporation, Tokyo, Japan) using Cu Kα radiation at a wavelength of λ = 0.154 nm. The performance of the resistance sensor and capacitive sensor was measured using a bench digital multimeter (Keysight 34465A, Keysight, Santa Rosa, CA, USA) at two-electrode resistance function and capacitance measurement function, respectively.

The electrical resistance of the various yarns was accurately determined using a bench digital multimeter, specifically the Keysight 34465A model. This measurement was facilitated by a custom-built four-point probe setup, which featured a precise interspacing of 2.54 mm between the probes. This method was used only for measuring the electrical conductivity of single fibers. The conductivity σ was calculated through:

(1) $$\sigma =\frac{L}{RS}$$

where *R*, *S*, and *L* are the resistance (Ω), the yarn cross-sectional area (cm^2^), and the length (cm) of the sample, respectively.

## 3. Results and Discussion

### 3.1. MXene Synthesis and Fiber Spinning

#### 3.1.1. MXene Synthesis with Controlled Sheet Sizes

The colloidal Ti_3_C_2_T_x_ MXene dispersion was prepared using the minimally intensive layer delamination (MILD) technique, as documented in prior research [33,34]. This method is known for its efficiency in exfoliating MXene layers with minimal disruption to their structure, resulting in high-quality dispersions suitable for various applications. As shown in Figure S1a, complete etching of Al layers from Ti_3_AlC_2_ MAX phase precursors yields highly delaminated MXene flakes. The successful delamination process in the synthesis of Ti_3_C_2_T_x_ MXene was evidenced by the elimination of the (104) peak from the X-ray diffraction (XRD) patterns of the MAX phase. This was further corroborated by the observed increase in interlayer spacing among the Ti_3_C_2_ units, as indicated by the downshift and broadening of the (002) peak, details of which are depicted in Figure S1c. These changes in the XRD patterns are indicative of the effective exfoliation of the material, confirming the structural transformation from the MAX phase to the desired MXene structure. The effect of filler size on the properties of the composite fiber was studied by using large (L-MX) and small MXene (S-MX) sheets. L-MX dispersion was obtained using as-prepared MXene. Average flake size of ~2 μm, was confirmed via dynamic light scattering (DLS) analysis and the representative atomic force microscopy (AFM) image shown in Figure S1d,e. To produce smaller flakes (S-MX), a certain volume of the same L-MX dispersion was probe-sonicated, which yields an average size of ~370 nm, as shown by its DLS profile in Figure S1d. Both L-MX and S-MX flakes possess a smooth surface and have a thickness of ~1.5 nm, confirmed via atomic force microscopy (AFM) images presented in Figure S1e,f. This measured flake thickness is higher than the reported value of ~1 nm obtained using TEM studies and predicted from DFT calculations [37,38], suggesting the presence of surface terminal groups and adsorbates on the nanosheet surface, such as water molecules.

#### 3.1.2. MXene/NR Composite Spinning Dopes

The hydrophilicity of Ti_3_C_2_T_x_ MXene allows its aqueous dispersion to be mixed with natural rubber latex, providing a simple and straightforward approach to prepare spinning dopes. Before mixing, the NR latex was prevulcanized using a conventional formulation as listed in Table S1. The prevulcanized natural rubber, also referred to as NR for simplicity, is prepared by partially crosslinking the latex by heating after drying, resulting in a 3D interconnected network of rubber particles. To obtain the spinning formulations, we blended the NS dispersion with aqueous Ti_3_C_2_T_X_ at various loadings from zero (pure NR) to 30 wt.% of Ti_3_C_2_T_x_ MXene (MX-30/NR). As shown in Figure S2, homogeneous MXene/NR dispersions were achieved, brought by the electrostatic repulsion between both negatively charged nanosheets and NR particles. These spinning dopes also showed a color gradient change from white NR latex to black, indicative of increased MXene loading.

#### 3.1.3. Wet-Spinning of MXene/NR Composite Fibers

The fabrication of MXene/NR composite fibers was carried out using the wet-spinning technique illustrated in Figure 1a. Here, the aqueous MXene/NR dope was continuously extruded into a coagulation bath to form the gel-state fiber, followed by subsequent vulcanization and drying steps in a hot oven at 100 °C for 5 min to form the crosslinked MXene/NR fiber. The comprehensive characterization of MXene/NR composite fibers involved a detailed examination of their morphological, electrical, and mechanical attributes. This analysis extended beyond merely assessing the impact of MXene loading and flake size; it also delved into the pivotal spinning parameters that dictate fiber formation. Among these, the choice of coagulation bath emerged as a critical factor, influencing the ultimate properties of the fibers. Such a methodical approach ensures a thorough understanding of how each variable contributes to the performance of the composite fibers. According to previous reports, both acetic acid and ethanol have proved to be a suitable coagulation bath for NR thread and MXene-based fibers [14,17,39]. Firstly, we chose ethanol as the coagulation bath to prepare MXene/NR fibers. We found that when the MXene loading exceeded 20 wt.%, the fibers broke easily during the collection process. To attain fibers with higher MXene loading, a concentrated acetic acid bath was used as the coagulation bath; however, the NR component of the dope suffered from quick solidification, forming a droplet on the top of the needle. After we assessed the spinnability of MXene/NR dope in various coagulation baths, the dilute acetic acid (V_AcOH_:V_water_ = 3:7) and ethanol (V_ETH_:V_water_ = 8:2) solutions were selected for fabricating continuous composites fibers, as they allow MXene loading in the range of 0–30 wt.%. As demonstrated below, in the electrical conductivity part, composite fibers spun in the AcOH bath exhibited better conductivity, so we selected AcOH as the coagulation bath in the scalable fiber spinning process. Figure 1a depicts the scalability of this spinning process, showing a 5 m-long continuous MXene/NR fiber produced using a spinning dope of 30 wt.% S-MX.

### 3.2. Fiber Characterizations

#### 3.2.1. Morphology Changes of Fibers

The evolution of fiber morphology upon varying MXene loading was assessed using scanning electron microscopy (SEM). Pure vulcanized NR fibers showed circular cross-sections and smooth surfaces (Figure 1c,d), without any distinct NR particles, implying sufficient stretching and crosslinking of NR chains between NR particles. Additionally, there is no observable difference in the microstructure of pure NR fibers even when using different coagulation baths, and both showed comparable average diameters of ~310 μm when extruded through a 23-gauge needle. Although MXene nanosheets appear to be dispersed homogeneously throughout the fibers, their surface became rough upon increasing MXene loading, as shown in Figure 1e and f (for S-MX-15/NR and S-MX-30/NR), and a similar trend was observed for various MXene/NR ratios, as summarized in Figure S2f–i. The average diameter of composite fibers also shrank (e.g., to ~198.3 μm for S-MX-30/NR fiber), which is because the low volume fraction of MXene within the composite fiber reduced the total mass of the fibers. At low MXene loading content, less distinct NR particles were observed, which means a natural rubber component had formed a continuous crosslinked phase (Figure 1e). However, this higher amount of MXene nanosheets caged the NR particles and resulted in poor crosslinking efficiency, which consequently led to more frequent fiber breakages (Figure 1g,h). The distribution of MXene within the samples was assessed through EDX maps of their cross-sections (S-MX-15/NR in Figure 1i). These maps confirmed the high amount of Ti_3_C_2_ MXene indicated by the even distribution of Ti, while the appearance of S and N represented the sulfur compound and protein coming from the NR latex. The homogeneous appearance of elements in these EDX maps collectively imply that all nanomaterial components in the vulcanized S-MX-15/NR fibers are well mixed and evenly distributed.

#### 3.2.2. Electrical Conductivity Test

**Figure 2:**
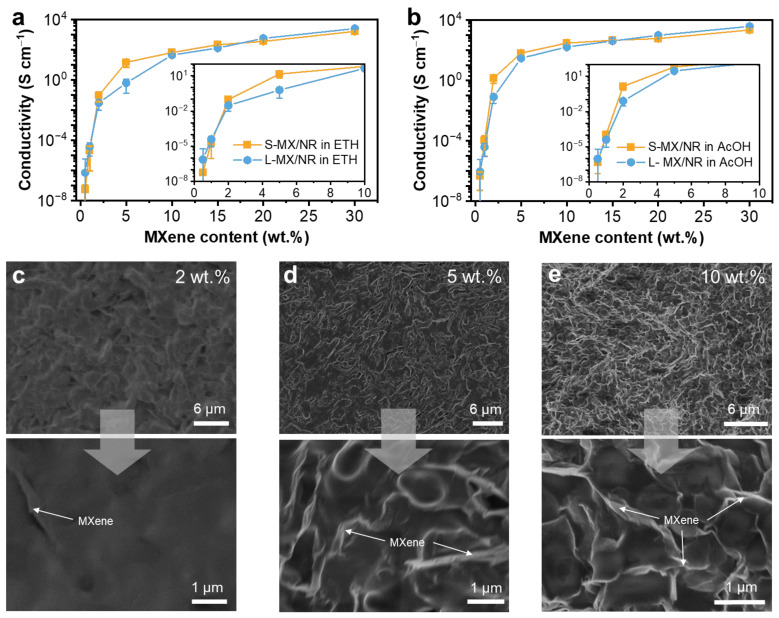
The electrical conductivity of MXene/NR fiber as functions of MXene contents in coagulation bath of (**a**) ethanol (ETH) and (**b**) acetic acid (AcOH). The SEM image of the cross-section of the MXene/NR fiber with increasing L-MX content from (**c**) 2 wt.%, to (**d**) 5 wt.%, and (**e**) 10 wt.%. The bottom magnified images show the distribution of MXene and NR.

In general, the percolation network of conductive fillers is critical for providing a conductive pathway in the composite matrix [40]. Benefitting from the planar geometry of MXene flakes, their composites have exhibited impressively low percolation thresholds. For example, conductive fibers were acquired for ~1 wt% for MXene/PU fiber spun in isopropanol [14], and ~3.6 wt% for spray-coated MXene/ink composites [41]. To obtain the percolation threshold of MXene, MXene contents were controlled from ~0.5 wt% up to 30 wt.% by using ethanol and acetic acid as coagulation baths separately and their respective electrical conductivities were measured (Figure 2). The conductivity (σ) of MXene/NR fibers was calculated from the length (*L*) against the fiber resistance (*R*) and cross-sectional area (*S*) of fibers. The overall conductivity values of NR fibers made from small and large MXene flakes were both higher when spun in the acetic acid bath than those in ethanol. This difference is caused by the faster coagulation rate in acetic acid compared with ethanol, which resulted in denser and closely packed fiber morphology. The conductivity of MXene/NR fibers also directly increased with higher MXene loading regardless of the coagulation bath. The highest electrical conductivity value was obtained for L-MX/NR fibers at 30 wt.% of large MXene sheets spun in the acetic acid bath (~3858 S cm^−1^). This value is 1.5 times higher than L-MX/NR fibers spun in the ethanol bath (~2555 S cm^−1^), 1.7 times higher than S-MX-30/NR fibers spun in the acetic acid bath (~2220 S cm^−1^) and 2.3 times higher than S-MX-30/NR fibers spun in the ethanol bath (~1654 S cm^−1^). Interestingly, we noticed that the conductivity values of L-MX/NR fibers at low (1 wt.%) and high (>20 wt.%) MXene loadings are both higher than their counterpart S-MX/NR fibers at the same MXene content. Counterintuitively, the conductivity of S-MX/NR fibers at 2~15 wt% MXene loading is conversely higher than the L-MX/NR fibers. This result indicates that large MXene sheets are capable of forming conductive networks even at low loading, which then results in better connections at high loading (Figure 2c–e). In contrast, S-MX tends to form conductive paths with an intermediate number of binders serving as bridging agents (Figure 1d–h), which become blocked when the flake content is too low, and a percolation network is no longer achievable (<1 wt.%).

#### 3.2.3. Mechanical Properties of MXene/NR Fiber

**Figure 3:**
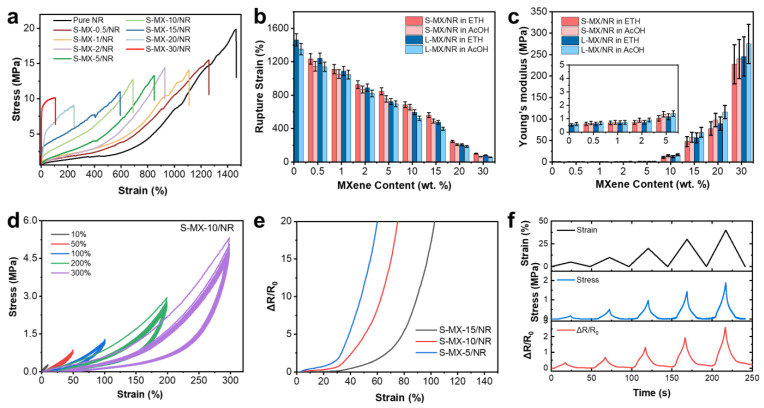
(**a**) Stress−strain curves of S-MX/NR fibers that spun in AcOH with different NR loading ratios. (**b**) The summarized Young’s modulus and (**c**) strain at break calculated from stress curves of fibers at various MXene loadings and spinning conditions. (**d**) The stretching cycles of S-MX-10/NR fiber at different strains for 20 cycles. (**e**) The resistance changes at increasing strains for S-MX-5/NR, S-MX-10/NR and S-MX-15/NR fibers. (**f**) The changes in resistance and stress of 1 cm-long S-MX-10/NR fiber during stretching–releasing cycles at different strains for motivation detection.

The mechanical properties of MXene/NR fibers spun in different coagulation baths and MXene contents were also evaluated. The typical stress–strain curves of S-MXene/NR fibers with increasing MXene loading (spun in ethanol) are plotted in Figure 3a. The NR fiber demonstrates exceptional super-elastic properties, characterized by a tensile strength of 19.6 MPa and an elongation at break of approximately 1460%. Both tensile strength and breaking strain, however, gradually decreased upon increasing of MXene loading. For S-MX-30/NR fibers spun in alcohol, the tensile strength and breaking strain dropped to less than 10.2 MPa and 100%, respectively. Three typical regions were also observed from strain–stress behavior of MXene/NR fibers, namely (1) the initial elastic/stiff response, (2) strain-induced softening and (3) strain-induced hardening [14]. For pure NR fiber, strength increased gradually due to the lower crystallizable at the early strain stage, then significantly boosted at around the 500% strain region, which can be attributed to the crystallization of rubber chains during stretching. In the MXene/NR composite fibers, the 2D Ti_3_C_2_T_x_ sheets serve as the rigid “bricks”, while the linearly structured NR chains fulfill the function of the organic “mortar”. The uniform packing of the “brick−mortar” structure has the capability to be tuned towards a certain level of mechanical integrity (e.g., increased stiffness by adding more of the high modulus component). The data presented in Figure 3b,c show a comparative analysis of strain at break and the Young’s modulus for various fibers under different MXene loadings and spinning conditions. The Young’s modulus calculated from stress–strain curves indicates how adding MXene significantly reinforces the soft NR matrix. Specifically, the strain at break for MXene/NR fibers shows a remarkable decrease from 1280% for S-MX-0.5/NR to less than 100% for S-MX-30/NR, which is attributed to MXene’s inherent stiffness. Concurrently, the Young’s modulus increased from approximately 0.53 MPa for pure NR fibers to about 227.6 MPa for S-MX-30/NR fibers. These findings highlight that incorporating stiff MXene fillers into NR fibers significantly provides robustness to the fiber structure.

Additionally, we noticed that the MXene/NR fiber spun in ETH showed higher rupture strain than that of fiber spun in AcOH, while fibers spun in AcOH exhibited slightly higher Young’s modulus. This difference suggests that the coagulation bath composition affects the fiber mechanical properties, which can be related to the MXene–rubber latex interface and coagulation mechanism, and this could be further studied. Considering that the fiber in the AcOH bath obtained slightly higher electrical conductivity, we finally decided to use the fibers in AcOH for study of its application. To investigate the behavior related to strain-induced softening and stress hysteresis, we conducted standard cyclic uniaxial strain sweep experiments on representative S-MX-10/NR composite fibers. Figure 3d summarizes the cyclic loading and unloading curves of S-MX-10/NR fiber, spun in AcOH and subjected to a consistent strain of at 10%, 50%, 100%, 200% and 300%, respectively. The cycling curve shows excellent repeatability, indicating a good cycling range for potential durable elastic sensors.

#### 3.2.4. Demonstration as a Strain Sensor

One behavior of MXene/NR fibers that is useful in practical applications is its strain-induced resistance change. As demonstrated in Figure 3e, fibers with varying MXene content, specifically S-MX-5/NR, S-MX-10/NR, and S-MX-30/NR, all exhibit a notable spike in resistance upon increasing strain. This resistance change is indicative of the fibers’ sensitivity to deformation, which is vital for applications in flexible electronics and smart textiles. The S-MX-5/NR fiber, however, displays a limited operational strain under 50%, suggesting that while it can detect small deformations, its utility may be restricted by this narrow range. In contrast, the S-MX-30/NR fiber shows a diminished resistance change at strains below 60%, indicating a higher tolerance for deformation before significant changes in resistance are observed. Additionally, the varying behaviors of these fibers under strain underscore the importance of tuning the MXene content to tailor the mechanical and electrical properties for specific end-use scenarios. For this application demonstration, we chose the S-MX-10/NR for the strain sensor study, which meets the criteria for a broader functional range. As shown in Figure 3f, the stress and resistance changes are highly aligned with the strain changes in the range of 5 to 40%, suggesting good sensitivity.

#### 3.2.5. X-ray Scattering Characterization of MXene/NR Fiber

**Figure 4:**
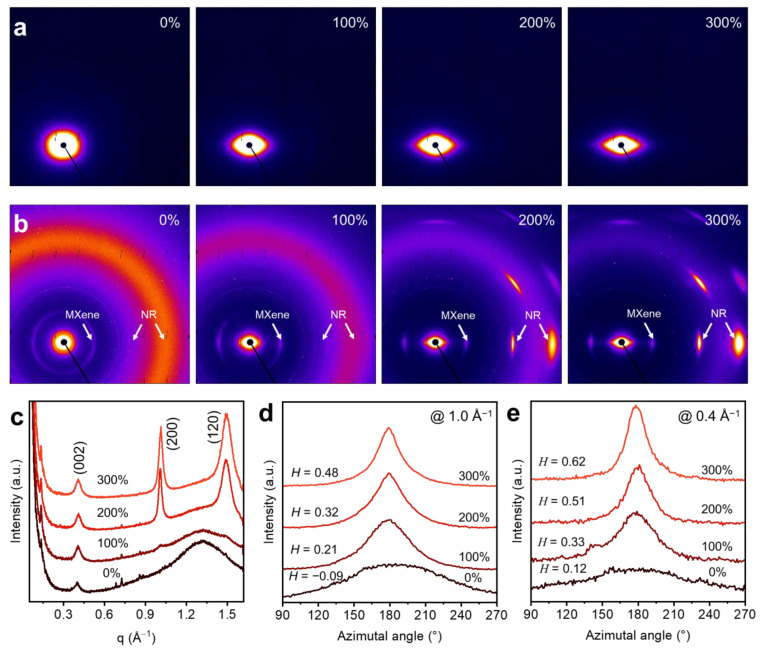
(**a**) 2D SAXS images of S-MX-10/NR fiber at increasing strain with data collected at a sample to detector distance of 1800 mm. (**b**) WAXS images of the MXene/NR composite fiber at increasing strain with data collected at a sample to detector distance of 100 mm. (**c**) 1D linear profiles of MXene/NR fiber along the radius direction (or meridian direction) between the azimuthal angle of 0° to 90°. (**d**) Azimuthal scan 1D profiles of the MXene/NR fiber at different strains and the *q* range is selected between 1.0 ± 0.5 Å^−1^, the *H* refers to the Hermans orientation factor. (**e**) Azimuthal scan 1D profiles of the MXene/NR fiber at different strains and the *q* range is selected at 0.4 ± 0.5 Å^−1^.

The data from Figure 4 provide a multifaceted view of the structural changes in S-MX-10/NR fiber under strain, utilizing both small-angle X-ray scattering (SAXS) and wide-angle X-ray scattering (WAXS) techniques. In Figure 4a, the 2D SAXS images captured at a sample to detector distance of 1800 mm reveal the nanostructural evolution of the S-MX-10/NR fiber as it undergoes increasing strain. With increasing strain, the shape of the SAXS pattern changed from circular to anisotropic, suggesting that polymer chains are aligned along the fiber direction at high strain. Figure 4b presents the WAXS images of the MXene/NR composite fiber, taken at a much closer sample to detector distance of 100 mm, which allows the observation of its crystalline structure as well as the degree of MXene alignment within the fiber under higher strains. By reducing the data into line profiles, we can identify that the peak at 0.4 Å^−1^ belongs to the layer stacking of MXene sheets (002 phase). The peaks at 1.0 Å^−1^ and 1.5 Å^−1^ are attributed to the (200) and (120) phases of highly oriented crystallites in the NR, where stronger crystallized spots are visible upon increasing the strain ratio (Figure 4c). To quantify the orientation changes of MXene and NR, the selected q range between at 1.0 ± 0.5 Å^−1^ and at 0.4 ± 0.5 Å^−1^ were plotted. Patterns in this q range correspond to structural features of NR and MXene, respectively. Here, we calculated the Herman’s orientation factor, denoted by *H*, which is a quantitative measure of the fiber’s orientation, where changes in *H* reflect the response in MXene sheet alignment under different strains. The Herman’s factor (*H*) is defined as:

(2) $$H=\frac{3<{cos}^{2}\Psi >-1}{2}$$

*H* = 1 for the orientation Ψ = 0° and *H* = −0.5 for Ψ = 90°. The definition is independent of the symmetry (works for two-fold or four-fold symmetries, or no symmetry at all). Both MXene sheets and NR polymer chains showed improved alignment as strain, as evidenced by the *H* value, increased from −0.09 to 0.48 (at 1.0 ± 0.5 Å^−1^) and from 0.12 to 0.62 (at 0.4 ± 0.5 Å^−1^).

### 3.3. Knitting of MXene/NR Fiber

**Figure 5:**
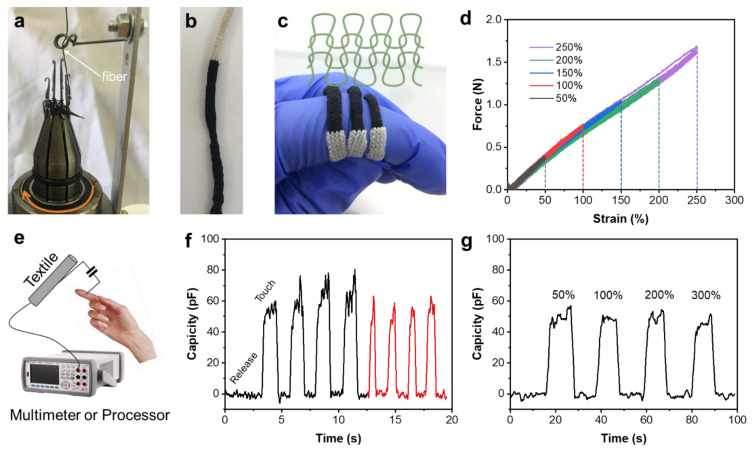
(**a**) Knitting process of the MXene/NR fiber captured in action. (**b**) The resulting tubular knitted textile. (**c**) A comparison of the tubular knitted textile that was produced at different strains of 10% (**left**), 50% (**middle**) and 100% (**right**). (**d**) The force–strain curve of the tubular knitted textile prepared at strain of 10%. (**e**) The scheme illustration of the textile-based capacitive touch sensor. (**f**) The capacitance signal changes at touch–release cycles with a touching period of ~2 s (black line) and ~1 s (red line). (**g**) The capacitance signal changes of the tubular knitted textile at different strains from 50% up to 300%.

Owing to the enhanced Young’s modulus, MXene/NR fibers possess sufficient strength to be woven into textiles, as depicted in Figure 5a,b. This process not only demonstrates the fibers’ adaptability to textile manufacturing but also their potential for commercial scalability. By adjusting the tension applied to the MXene/NR fiber during knitting, we noticed that the textile diameter reduced as tensile strength increased (Figure 5c). This characteristic is crucial for wearable technologies where flexibility and durability are paramount. The force–strain curve in Figure 5d, corresponding to a strain of 10%, provides insight into the textile’s mechanical properties, indicating a robust yet flexible material capable of withstanding external forces without compromising its form or function. When fibers are knitted into a textile, the inter-fiber connections can introduce variability in the electrical properties. In the case of our circular knitted textile, we did not measure the electrical conductivity in the same manner as for single fibers. Instead, we used the conductive textile for capacitive touch sensors. The schematic on the textile-based capacitive touch sensor (Figure 5e) underlines the innovative integration of MXene/NR fibers in electronic devices. The sensor’s responsiveness to touch, as shown in Figure 5f, with signal changes recorded over touch–release cycles of approximately two seconds and one second, suggests a high degree of sensitivity and rapid signal recovery, making it suitable for interactive applications. The methodology involves applying a controlled strain to the sensor while monitoring the capacitance to identify any changes that occur due to deformation. This process is critical for understanding the sensor’s sensitivity and operational limits, which are essential for ensuring reliable performance in practical applications. Figure 5g highlights the textile’s capacitive behavior under different strains, ranging from 50% to 300%. When the textile was under tension, a dry finger touched the surface of the textile, which showed a consistent signal of the capacitance change around 55 pF. The consistent signal change across this wide range of strains underscores the textile’s potential as a reliable sensor in various deformation states, which could be particularly beneficial in monitoring applications that require detecting and measuring dynamic movements.

Not limited by this study, we proposed that the exploration of thermal and moisture stability, along with sensitivity to environmental factors such as humidity, represents a frontier in the field of sensor research. For the broader scientific community, delving into these areas is not just beneficial but essential for the advancement of sensor technology. The robustness of sensors against varying temperatures and moisture levels will be a defining factor in their applicability across diverse industries and climates. Furthermore, the fatigue performance of sensors is a critical parameter that all researchers must scrutinize. It is imperative to ensure that sensors can withstand the rigors of long-term use without degradation in performance, also considering the impact of different knitting configurations. This will pave the way for more reliable and durable sensing solutions. Scalability and commercialization are also pivotal considerations for the field. Researchers must navigate the challenges of transitioning from laboratory prototypes to market-ready products. This includes optimizing manufacturing processes, ensuring cost-effectiveness, and meeting industry standards. In summary, the collective efforts of the research community should focus on enhancing sensor stability, durability, and commercial feasibility. These efforts will not only contribute to the body of knowledge but also catalyze the development of innovative sensor technologies that can be seamlessly integrated into everyday life and industrial applications.

## 4. Conclusions

The comprehensive analysis of MXene/NR fibers elucidated the relationship between the microstructure of MXene/NR fibers and their macroscopic mechanical properties. Understanding this relationship is shown to be key in optimizing the performance of MXene/NR composite fibers for potential applications in areas such as flexible electronics, smart textiles, and advanced composites where both mechanical strength and flexibility are required. The data indicate that both the content of MXene and its dispersion throughout the NR matrix are critical factors influencing the fiber’s strain response. This insight could be utilized to engineer fibers with customized properties for designated applications.

## Data Availability

The original contributions presented in the study are included in the article/supplementary material, further inquiries can be directed to the corresponding author/s.

## Supplementary Materials

The supporting information can be downloaded at: https://www.mdpi.com/article/10.3390/polym16131824/s1. Figure S1. Scanning electron microscopy (SEM) images of (a) Ti3AlC2 (MAX phase) and (b) delaminated Ti_3_C_2_T_x_ (MXene) flakes. (c) X-ray diffraction (XRD) patterns of Ti3AlC2 MAX powder (black curve) and Ti_3_C_2_T_x_ MXene film (blue curve). Atomic Force Microscopy (AFM) images and height profile of (d) L-MX nanosheet and (e) S-MX nanosheet. (f) Size distribution of S-MX and L-MX flakes in water obtained with dynamic light scattering (DLS) technique. Figure S2. (a) The photo of Ti_3_C_2_T_x_ MXene/NR dispersions and (b–f) SEM images of S-MX/NR composites fibers with different MXene loading amount. The scale bars are 60 μm. Table S1. Formulation of the prevulcanized NR compound.
