# Supplementary material for: Knitting Elastic Conductive Fibers of MXene/Natural Rubber for Multifunctional Wearable Sensors
Source: Polymers (Basel). 2024 Jun 27;16(13):1824. doi: 10.3390/polym16131824 (PMC11244089; doi:10.3390/polym16131824)
Supplement: Supplementary file 1 [file polymers-16-01824-s001.zip › polymers-3051163-supplementary.pdf]

polymers

MDPI

Supplementary Materials

Knitting Elastic Conductive Fibers of MXene/Natural Rubber for Multifunctional Wearable Sensors

Zirong Luo [1], Na Kong [1,2], Ken Aldren S. Usman [3], Jinlong Tao [1,*], Peter A. Lynch [3], Joselito M. Razal [3] and Jizhen Zhang [1,3,*]

Experimental

Table S1. Formulation of the prevulcanized NR compound.

Ingredients	Parts by weight (dry)	Parts by weight (wet)
60% Natural rubber latex	600	1000
50% Sulfur dispersion	0.9	1.8
50% Zinc oxide dispersion	0.5	1.0
50% Accelerator ZDC dispersion	0.8	1.6
20% Peregal O dispersion	0.5	2.5
10% Potassium hydroxidesolution	1.5	8.0

Characterization

A scanning electron microscope (HITACHI S4800) was used to study the morphology and the cross-section of the as-spun CNT yarns. Atomic force microscopy (FlexAFM with software of Nanosurf C3000) was used to obtain images under air tapping mode to measure the thickness of MXene flake. AFM samples were prepared by drop-casting diluted aqueous dispersion on silicon wafers. The mechanical properties of the yarns were measured using a universal tensile testing system (Tensile Tester TM2101-T5) with a 50 N load cell. Yarn samples were fixed between the grips with the aperture size of 1 cm and strain and stress data were recorded at the extension rate of 1 mm min^{-1}. Zeta potential of $T_3C_2T_x$ MXene solution with different NRL loading was measured using a Malvern Zetasizer Nano ZS90, which is also used for dynamic light scattering (DLS) to measure the size distribution of $T_3C_2T_x$ MXene and NR particles. The X-ray diffraction (XRD) patterns of the MAX phase, MXene and various MXene films were obtained using a powder diffractometer (Rigaku SmartLab 9 kW) with Cu Kα radiation (λ=0.154 nm).

The electrical resistance of the different yarns was measured using a bench digital multimeter (Keysight 34465A) by the aid of a custom-built four-point probes with inter-spacing of 2.54 mm. The conductivity σ was calculated through:

$$\sigma = \frac{L}{RS} \quad (1)$$

where R, S, and L are the resistance (Ω), the yarn cross-sectional area (cm^2), and the length (cm) of the sample, respectively.

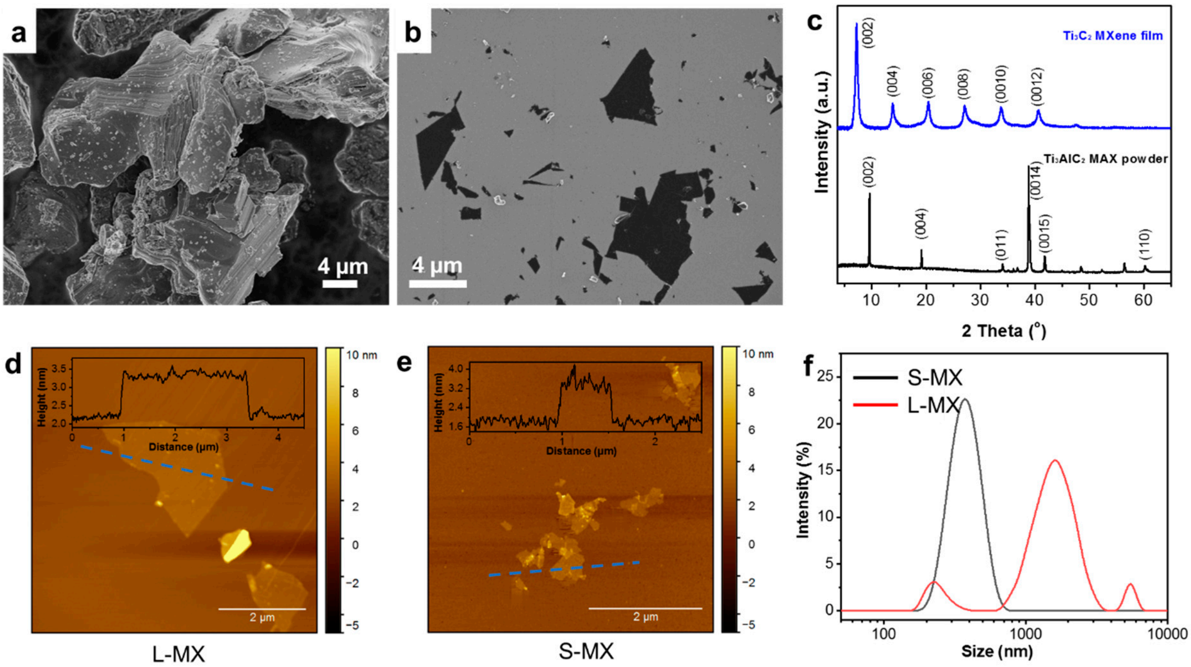

Figure S1. Scanning electron microscopy (SEM) images of (a) Ti_3AlC_2 (MAX phase) and (b) delaminated $Ti_3C_2T_x$ (MXene) flakes. (c) X-ray diffraction (XRD) patterns of Ti_3AlC_2 MAX powder (black curve) and $Ti_3C_2T_x$ MXene film (blue curve). Atomic Force Microscopy (AFM) images and height profile of (d) L-MX nanosheet and (e) S-MX nanosheet. (f) Size distribution of S-MX and L-MX flakes in water obtained with dynamic light scattering (DLS) technique.

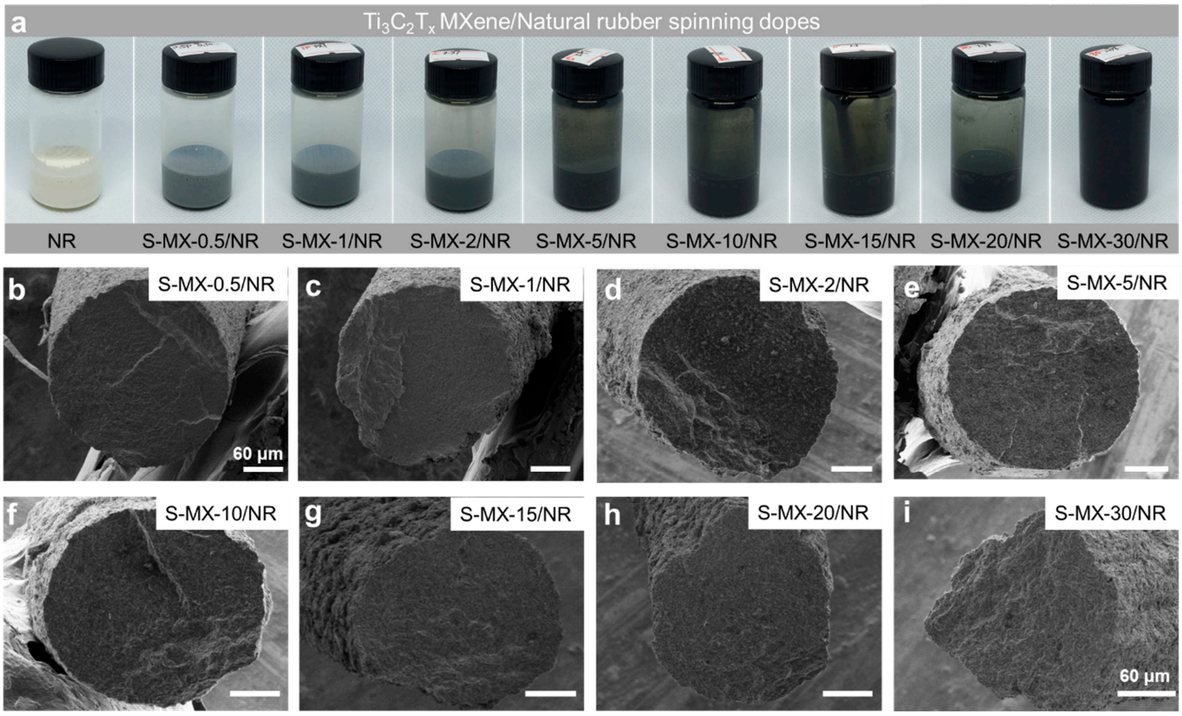

Figure S2. (a) The photo of $Ti_3C_2T_x$ MXene/NR dispersions and (b-f) SEM images of S-MX/NR composites fibers with different MXene loading amount. The scale bars are 60 μm.
